# Cost-effectiveness analysis of Anaprazole versus Ilaprazole for the treatment of duodenal ulcers in China

**DOI:** 10.3389/fphar.2024.1407435

**Published:** 2024-06-07

**Authors:** Huitong Ni, Jiaqi Shi, Ming Hu, Naitong Zhou, Shu Yang

**Affiliations:** West China School of Pharmacy, Sichuan University, Chengdu, China

**Keywords:** duodenal ulcers, Anaprazole, Ilaprazole, anchored matching-adjusted indirect comparison, cost-effectiveness analysis

## Abstract

**Objective:**

Anaprazole, an innovative drug, has shown promise in initial clinical trials for patients with duodenal ulcers (DU) in China. This study aimed to evaluate the potential effects, safety, and cost-effectiveness of Anaprazole compared to Ilaprazole in the treatment of DU and the budgetary impact on the healthcare system.

**Methods:**

Two multicentre, randomized controlled trials were used as data sources. The efficacy and safety of Anaprazole and Ilaprazole were compared using an anchored matching-adjusted indirect comparison (MAIC). A cost-utility analysis (CUA) was performed using a Markov model. A budget impact analysis (BIA) was conducted to evaluate the impact on the expenditure of the Chinese healthcare system. Deterministic and probabilistic sensitivity analyses were undertaken to test the uncertainty.

**Results:**

The study findings indicated that Anaprazole and Ilaprazole have similar efficacy and safety in treating DU (OR = 1.05; 95% CI, 0.94–1.01; *p* = 0.35; OR = 0.63; 95% CI, 0.39–1.08; *p* = 0.12). The ICUR was 2,995.41¥/QALY, which is below the WTP threshold. The CUA results showed that Anaprazole is a cost-effective intervention with a probability of 85% at a given threshold. The results demonstrated strong robustness in the sensitivity analysis. Anaprazole imposed a low burden on the Chinese healthcare budget in the BIA.

**Conclusion:**

Compared with Ilaprazole, Anaprazole has similar efficacy, safety, and high cost-effectiveness, while also impacting the total expenditure of the healthcare system.

**Clinical Trial Registration:**

ClinicalTrials.gov, identifier NCT04215653 and NCT02847455

## Introduction

Approximately 5%–10% of the global population suffers from peptic ulcer disease (PUD), with an annual incidence rate of about 0.1%–0.3% (Tarasconi et al., 2020). According to the Global Burden of Disease (GBD) 2019 statistics, PUD in China has a higher incidence rate, mortality, and Disability-Adjusted Life Years (DALYs) compared to other countries (GBD, 2020). Peptic ulcer mainly includes two types of duodenal ulcer and gastric ulcer, among which duodenal ulcer (DU) and gastric ulcer (GU) occur in a ratio of about 3:1. DU is a common chronic disease characterized by defects in the mucosal and muscular layers of the duodenum (Lanas and Chan, 2017). It can occur at any age but is most common between the ages of 20 and 50, with a higher prevalence in males (Levenstein et al., 1997). DU often presents with recurrent attacks, especially during seasonal changes. Symptoms include severe abdominal pain, and in severe cases, complications such as bleeding, perforation, and obstruction may occur. The severity of symptoms is directly related to the extent of the ulcer. The main causes of DU include abnormal gastric acid secretion, *Helicobacter pylori* infection, and the use of non-steroidal anti-inflammatory drugs (NSAIDs) and aspirin (Chan and Leung, 2002).

Gastric acid suppression therapy is an important component of the current treatment plan for DU. Proton pump inhibitors (PPIs) are a mainstay of peptic ulcer treatment that act by irreversibly inhibiting H+/K +- ATPase. They are strongly recommended as the first-line treatment for acid suppression and the treatment of peptic ulcers in guidelines and consensus statements such as the “Evidence-based Clinical Practice Guidelines for Peptic Ulcers (2020)” in Japan (Kamada et al., 2021).

Anaprazole sodium enteric-coated tablet (Anaprazole) is a domestically developed PPI in China, with international patent. In June 2023, Anaprazole was approved as a class 1 of innovative drugs in China for the treatment of duodenal ulcer based on a multicenter randomized controlled trial (RCT) (ClinicalTrials.gov NCT04215653) and has been included in the updated National Reimbursement Drug List (NRDL, 2023). It inhibits gastric acid secretion and is used in the treatment of DU. Compared to other PPIs that rely mainly on CYP2C19 enzyme metabolism, Anaprazole achieves co-metabolism through targeted structural design, involving multiple enzymes (7 CYP enzymes) and non-enzyme pathways. The main metabolic enzyme is CYP3A4, similar to llaprazole, but its contribution rate is only 24.6% (Liu et al., 2023). This makes Anaprazole a potentially safer choice for elderly patients and other populations who are using multiple medications.

Currently, the economics of Anaprazole as a new generation of PPI drugs have not been effectively evaluated. Under the background of rising medical costs and limited medical resources, pharmacoeconomics is helpful to reasonably control the rise of drug costs and optimize the allocation of medical resources (Dai et al., 2024). According to recommendations of guideline (Liu et al., 2020), Ilaprazole enteric coated tablet (Ilaprazole) was selected as the reference drug because of the same indication and extensive clinical use. As the first novel PPI in development for peptic ulcer treatment, Anaprazole has the advantages of multi-enzyme plus non-enzyme metabolism, intestinal and renal double-channel excretion, etc., which can provide a safer choice for patients with multiple drugs and renal dysfunction. Furthermore, Anaprazole is based on data from the entire Chinese population and has shown a good safety profile in both Chinese duodenal patients and healthy individuals (Wang et al., 2024).

Although both Anaprazole and Ilaprazole are new generation PPI drugs, Ilaprazole was launched earlier in China (2007) and occupied a large market share in the Chinese market. Compared with the western market, China has a higher incidence of duodenal ulcer. Anaprazole RCT trial is conducted based on the data of the whole Chinese population. By comparing the safety, effectiveness of Anaprazole and Ilaprazole, it may provide a new clinical treatment option suitable for the Chinese population. Additionally, conducting an economic analysis of Anaprazole will assess its impact on the Chinese healthcare system. Understanding the cost-effectiveness and affordability of Anaprazole is vital for healthcare decision-makers to evaluate its financial implications and ascertain its value relative to other treatments. Such evaluations are crucial for informed resource allocation and policy-making, enhancing the sustainability and efficiency of healthcare in China.

## Materials and methods

### Interventions

This study employed a multicenter RCT comparing Anaprazole versus Rabeprazole (Zhu et al., 2022) (ClinicalTrials.gov, NCT04215653) and another RCT comparing Rabeprazole versus Ilaprazole (Li et al., 2019) (ClinicalTrials ID: NCT02847455) as sources of clinical data. The experimental group comprised 220 patients using Anaprazole, while the control group included 129 patients using Ilaprazole.

Anaprazole is available in tablet form, with a dosage of 20 mg per tablet. In the experimental group, patients were instructed to take one 20 mg tablet of Anaprazole orally once daily before breakfast for a duration of 4 weeks.

Ilaprazole, also in tablet form, comes with a dosage of 5 mg per tablet. In the control group, patients were instructed to take two 5 mg tablets of Ilaprazole orally once daily before breakfast for a duration of 4 weeks.

### Comparison of efficacy and safety

**FIGURE 1:**
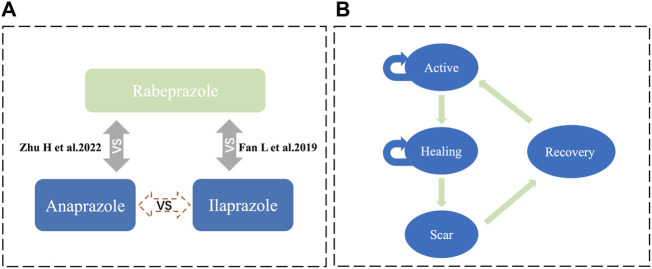
Model structure **(A)** illustrates the MAIC analysis framework, facilitating an indirect comparison between Anaprazole and Ilaprazole using Rabeprazole as a bridge. **(B)** depicts the Markov model.

Adjusted indirect comparisons are recommended by various decision-making bodies and guidelines for studies lacking head-to-head clinical trials (Phillippo et al., 2018). Given the absence of direct comparative RCTs between Anaprazole and Ilaprazole, an anchored MAIC was conducted to analyze the effect and safety between the two treatments. This approach, as depicted in Figure 1, accounts for confounding factors and provides a more reliable comparison than unadjusted indirect comparisons.

Data from the Ilaprazole control group were matched by weighting individual case data from multicenter clinical trials of Anaprazole. Using a logistic regression model to calculate propensity scores and performing inverse probability weighted matching, the baseline distribution between groups reached equilibrium, allowing for efficacy and safety comparisons within a study population with balanced baseline distributions post-matching (Liu, 2022). Using Bucher’s indirect comparison method (Bucher et al., 1997), the relative efficacy differences between the treatment regimens were quantified: the effect of Anaprazole vs. Rabeprazole was represented as *logOR*_AC_, the effect of Ilaprazole vs. Rabeprazole as *logOR*_BC_, and the effect of Anaprazole vs. Ilaprazole (*logOR*_AB_), which is calculated by Eq. 1:

$${\text{logOR}}_{\text{AB}}={\text{logOR}}_{\text{AC}}\;‐\;{\text{logOR}}_{\text{BC}}$$ (1)

SPSS 18.0^®^ was utilized to process the data, with count data expressed in percentages and analyzed using the χ^2^ test. Measurement data are presented as x ± s and analyzed using the *t*-test, where *p* ≤ 0.05 indicates a statistically significant difference. The outcome indicators for binary variables in MAIC analysis are represented by odds ratio (OR), and analyzed using R^®^ 4.3.0 software to evaluate the effectiveness and safety of the Anaprazole group.

### Model description and structure

This study employed short-term simulations of patient health status transitions using a Markov model from the perspective of the Chinese healthcare system, implemented in Excel Microsoft (Figure 1). The Markov model encompassed four disease states: active (stage A), healing (stage H), scar (stage S), and recovery, aligned with relevant diagnostic criteria (Chun et al., 2014). The probabilities of transitioning between states were calculated using clinical trial data for Anaprazole and clinical literature for Ilaprazole. The cycle length was set at 2 weeks, with the study extending over 24 cycles.

The assumptions of this model included: (Tarasconi et al., 2020): Drawing from clinical trials and an anchored MAIC, 179 patients were included in the Anaprazole group and 139 in the Ilaprazole group, with all patients initially in Stage A at the time of model entry, transitioning through treatment phases until recovery. (GBD, 2020). Given the short cycle length, mortality was not considered; however, the recurrence rate within 6 months was included. (Lanas and Chan, 2017). It was assumed that patients in the active phase must progress to the healing phase before advancing to the scar phase, rather than moving directly to the scar phase.

### Data sources

#### Clinical efficacy

This study employed clinical research outcome indicators as measures of effectiveness. The primary clinical outcome indicator was the ulcer healing rate (Eq. 2), assessed using endoscopic images after 4 weeks of treatment. Efficacy evaluation criteria: patients were assessed via endoscopic images after 4 weeks of medication to determine the transition of ulcer status from stage A to stage S, which was defined as healing.

$$\text{Ulcer healing rate }\left(\%\right)=\left(\text{number of healing cases}/\text{total number of cases}\right)\times 100\%$$ (2)

#### Safety indicators

The incidence rate of ADRs was used as a safety evaluation parameter (Eq. 3).

$$\text{ADR incidence rate }\left(\%\right)=\left(\text{number of adverse reaction events}/\text{total number of participants}\right)\times 100\%$$ (3)

#### Resource use and costs

The pharmacoeconomic evaluation primarily considered direct medical costs, direct non-medical costs, indirect costs, and intangible costs (Liu et al., 2020). This study adopted a health system perspective, focusing exclusively on direct medical costs (Table 1). These included costs for drug treatment, registration fees, laboratory tests, and expenses related to adverse reactions (Garg et al., 2022). The price of Anaprazole was provided by the sponsoring enterprise, while the price of Ilaprazole was sourced from the local market (Yaozhinet, 2016). The pricing of other PPIs in the Chinese market was considered in the BIA (18). The total cost was calculated based on the frequency and dosage described in the medication guidelines and the actual circumstances of the clinical trials. Charges for registration and testing were derived from the price list of public medical services available on the hospital’s official website during clinical trials. Where hospitals lacked a public pricing list, prices from medical service lists at comparable hospitals within the same province were used.

**TABLE 1:** Parameter distributions.

Parameter	Baseline	Scope		Standard error^a^	Distribution
		Lower	Upper		
cost/¥					
Anaprazole treatment drug cost (20 mg)	27.2500	24.5250	29.9750	1.3903	Gamma
Ilaprazole treatment drug cost (5 mg)	13.0600	11.7540	14.3660	0.6663	Gamma
Examination cost	467.7675	420.9908	514.5443	23.8657	Gamma
Gastroscopy Examination Cost	606.5550	545.8995	667.2105	30.9467	Gamma
utilities/QALYs					
A stage	0.7030	0.6327	0.7733	0.0359	Beta
H stage	0.7790	0.7011	0.8569	0.0397	Beta
S stage	0.8420	0.7578	0.9262	0.0430	Beta

^a^ Standard deviation value of the parameter = (upper limit—lower limit) ÷ (2 × 1.96).

#### Health state utilities

A quality-adjusted life year (QALY) is an index used to measure the effects in this model and was calculated by multiplying life expectancy with health preference utility (Hu et al., 2022). The QALYs for each cycle represent the sum of the QALYs for each health state, calculated as the product of the number of life years for each state and the corresponding quality of life weight (Eq. 4) (Freath et al., 2022). Owing to the absence of utility data in the clinical trials, health utility values in this study were derived from a Markov model pharmacoeconomic analysis of PUD, (Xie and Tang, 2022), based on the European Five Dimensional Health Scale. In this study, the health utility values for the active, healing, and scar stages were 0.7030, 0.7790, and 0.8420, respectively (Table 1).

QALYs=number of life years×sum of the quality of life weights (4)

#### Discount rate

Guidelines (Liu et al., 2020) explicitly require the discounting of future costs and health outcomes when the research period exceeds 1 year. Due to the short duration of this study (less than 1 year), discounting was not applied.

#### Transition probabilities

The transition probabilities, representing the likelihood of patients moving from one treatment state to another, were estimated from various sources. For the Anaprazole group, probabilities were derived from individual patient data (Study Details, 2022), with a transfer probability of 0.9253 from the active phase to the healing phase and 0.9661 from the healing phase to the scar phase. For the Ilaprazole group, these probabilities were obtained from clinical studies (Ho et al., 2009), with a transfer probability of 0.8957 from the active phase to the healing phase and 0.9344 from the healing phase to the scar phase. The recurrence rate (the transition probability from the recovery stage back to stage A) was sourced from a large-scale, multicenter prospective cohort study on long-term follow-up of *H. pylori* reinfection (Xie et al., 2020), reported at 0.13%.

### CUA

This study conducted a CUA for economic evaluation. Published RCTs have documented short-term treatment effects for patients over 4 weeks, yet duodenal ulcers entail a certain degree of recurrence probability. To assess the long-term effects of treatment, this study conducted a CUA for economic evaluation. The incremental cost-utility ratio (ICUR) served as the principal outcome measure (Eq. 5), compared against the willingness-to-pay (WTP) threshold to assess the cost-effectiveness of the interventions. The WTP threshold was set at 1–3 times the *per capita* gross domestic product (GDP), representing the amount willing to be paid for each QALY (9). If the ICUR is less than the WTP, the intervention is considered cost-effective. This study used one times the *per capita* GDP of China in 2022 as the WTP threshold, amounting to 85,698¥/QALY.

$$\text{ICUR}=\left({\text{Cost}}_{1}‐{\text{Cost}}_{2}\right)/\left({\text{QALY}}_{1}‐{\text{QALY}}_{2}\right)$$ (5)

### Sensitivity analysis

Pharmacoeconomic evaluations using model methods often encounter uncertainties, including parameter and model uncertainty. To manage these uncertainties, this study utilized deterministic sensitivity analysis (DSA) and probability sensitivity analysis (PSA). In DSA, each cost parameter was varied within a range of ± 10% to assess its impact on the outcomes, depicted through a cyclone chart which visually represents the influence of multiple uncertain factors. PSA involved 1,000 second-order Monte Carlo simulations to sample the distribution of parameters. The results of PSA were displayed in incremental cost-utility plane scatter plots and cost-utility acceptance curves (CUAC), offering insights into the uncertainties of the results and aiding decision-makers in understanding the range of potential outcomes. These sensitivity analyses facilitated a more comprehensive evaluation of the pharmacoeconomic implications of the interventions.

### BIA

This study assessed the potential impact of Anaprazole on the health insurance fund from the perspective of health insurance administrators and decision-making departments over the next 3 years following its inclusion in the China National Reimbursement Drug List (NRDL) (Sullivan et al., 2014), using 2023 as the baseline year. The analysis included all PPIs drugs in the Chinese market as potential substitutes for Anaprazole. Target population parameters were derived from peptic ulcer epidemiology literature (Zhang, 2022) and the *GBD database* (GBD, 2019). Market share and cost data were sourced from the China Medical Economic Information Network (MENET) (MENET, 2022). China’s health insurance fund budget was reported on the official website of National Healthcare Security Administration (National Healthcare, 2022). The projected expenditures of the Chinese health insurance system on PPIs drugs from 2024 to 2026 were calculated to assess the impact of Anaprazole on health insurance budgets.

## Results

### Basic results

#### Baseline characteristics of patients included in MAIC analysis

Table 2 presents the baseline comparisons between the Anaprazole and Ilaprazole groups. Prior to matching, both groups exhibited similar distributions in age, gender, ulcer stage, and *H. pylori* infection rate. However, the proportion of patients with a single ulcer was lower in the Anaprazole group than in the Ilaprazole group. An indirect comparison was performed using anchor matching adjustment based on clinical ulcer characteristics, including age distribution, sex ratio, number of ulcers, and their location and rhythmicity. Following matching, the baseline characteristics of the two groups reached equilibrium.

**TABLE 2:** Baseline matching results.

Parameter	Ilaprazole N = 129	Before matching		After matching	
		Anaprazole N = 220	*p-value*	Anaprazole N = 179	*p-value*
Age (years), mean ± SD	37.64 ± 11.14	40.9 ± 11.10	0.476	37.64 ± 11.14	*p* > 0.05
Male sex	91 (70.54)	140 (63.63)	0.188	126 (70.54)	
Duodenal ulcer stage A1	94 (72.87)	181 (82.30)	0.152	130 (72.87)	
*Helicobacter pylori* positive	99 (76.74)	179 (81.36)	0.301	137 (76.74)	
Number of ulcers is 1	93 (72.09)	181 (82.27)	0.025	129 (72.09)	

#### Effectiveness comparison

**FIGURE 2:**
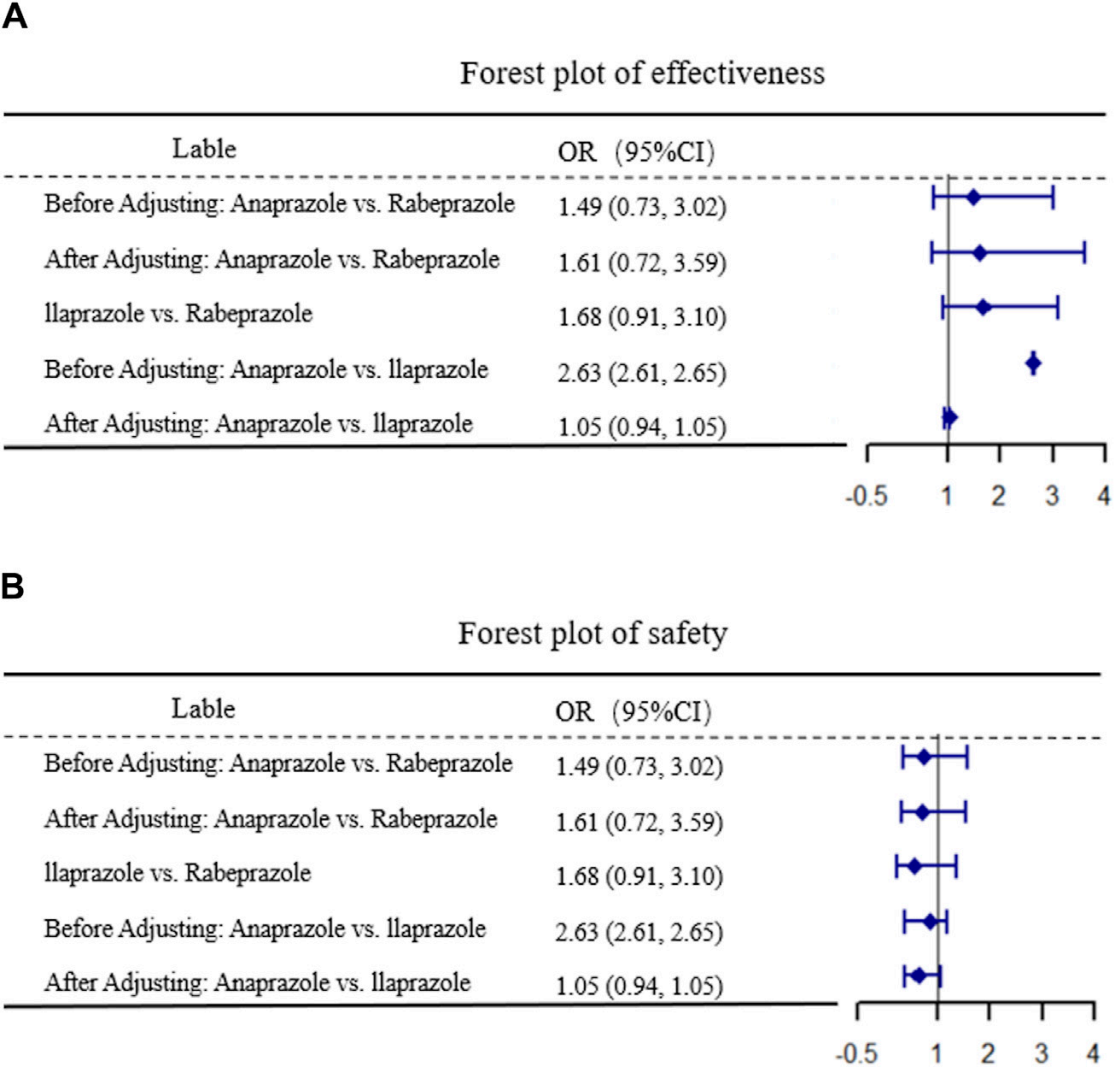
Results of MAIC. **(A,B)** display the comparative results of efficacy and safety, respectively.

After 4 weeks of treatment, the ulcer healing rates in the matched Anaprazole and Ilaprazole groups were 87.9% and 83.7%, respectively. The effectiveness of Anaprazole for duodenal ulcer (DU) treatment was comparable to that of Ilaprazole, with an OR of 1.05, 95% CI [0.94, 1.01], and *p* = 0.35, as illustrated in Figure 2.

#### Safety comparison

The incidence of ADR after 4 weeks was 8.21% in the Anaprazole group and 6.98% in the Ilaprazole group. The safety of Anaprazole in treating DU was comparable to Ilaprazole, with an OR of 0.63, 95% CI [0.39, 1.08], and *p* = 0.12, as depicted in Figure 2.

#### CUA

The results of the CUA, shown in Table 3, indicate that the composition and utility value of the Anaprazole group were higher than those of the Ilaprazole group. The ICUR was 2,995.41¥/QALY, which is below the WTP threshold. Thus, Anaprazole is deemed a cost-effective intervention compared to Ilaprazole at the established payment threshold.

**TABLE 3:** Results of ICUR.

Group	Cost (C, ¥)	Utility (U, QALYs)	C/U (¥/QALY)	ICUR (¥/QALY)
Anaprazole	5,423.61	0.9803	5,532.83	2,995.41
Ilaprazole	5,421.18	0.9794	5,534.93	

### Sensitivity analysis

#### Deterministic sensitivity analysis (DSA)

**FIGURE 3:**
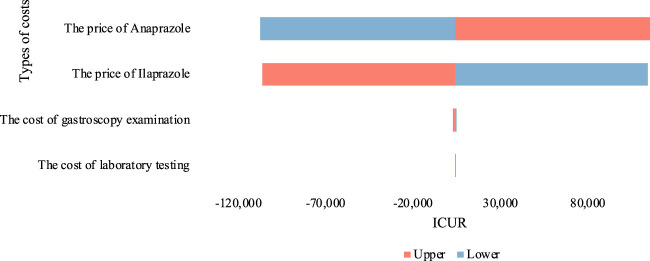
Results of sensitivity analysis. This figure displays the tornado diagram of DSA.

By assigning the cost parameters of both treatments to a range of ± 10% from the baseline value, we conducted a single-factor sensitivity analysis and depicted the outcomes using a tornado chart (Figure 3). The results were somewhat sensitive to the price parameters of Anaprazole and Ilaprazole. Despite fluctuations in drug prices, the ICUR value remained approximately equal to one time the GDP *per capita*, indicating robustness in our baseline analysis.

#### Probability sensitivity analysis (PSA)

**FIGURE 4:**
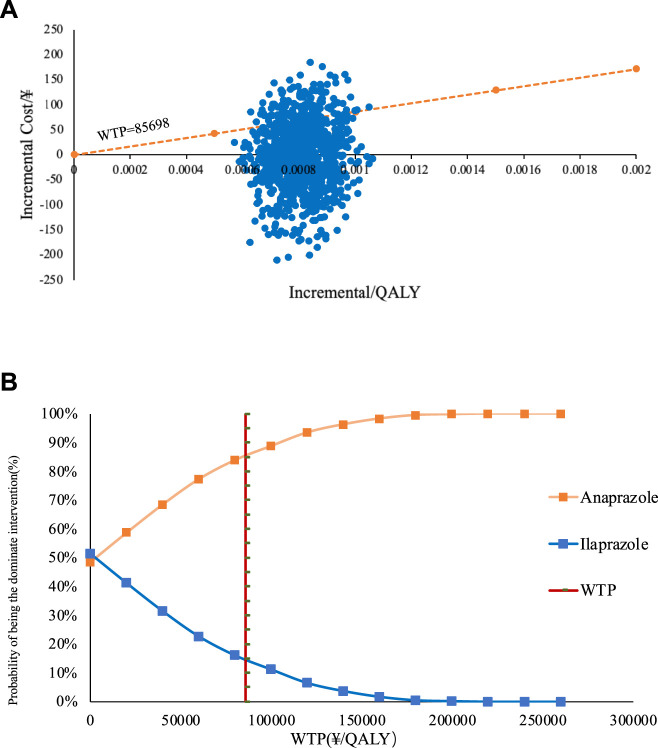
**(A)** The straight line in the graph represents the WTPs when the scatter of Anaprazole falls in the lower right of the line it means that the Anaprazole is economical compared to Ilaprazole at that WTP. **(B)** depicts the cost-effectiveness acceptability curve (CEAC).

The cost-utility scatter plot, presented in Figure 4, shows that most PSA values fall below one time the GDP *per capita*. This suggests that at a WTP threshold equal to one time the GDP *per capita*, Anaprazole has a higher probability of being economically viable compared to Ilaprazole. The economic justification for treating DU with Anaprazole increases with the WTP threshold. When the WTP is twice the GDP *per capita*, the likelihood that Anaprazole is cost-effective rises to 85%. The PSA findings align with the baseline analysis.

### BIA

In Scenario 1, where Anaprazole was not included in the NRDL, the projected annual Medicare fund expenditures for treating DU from 2024 to 2026 were ¥228.771 million, ¥226.274 million, and ¥238.034 million, respectively. In Scenario 2, assuming Anaprazole sodium enteric-coated tablets are included in the NRDL, the expenditures remain unchanged. The incremental costs represent 0.001957%, 0.003205%, and 0.005247% of the total budget of the China Health Insurance Fund, respectively. Factors such as consultation rate, prevalence of duodenal ulcer patients, and market share of PPI drugs may influence these results.

## Discussion

Research has pinpointed the diminished protective capacity of the gastric mucosa, excessive gastric acid secretion, and *H. pylori* infection as the main causative factors of peptic ulcers (el-Omar et al., 1995). Clinically, PUD is treated with a regimen combining PPIs, gastrointestinal mucosal protectors, and antibiotics. PPI is the first choice for the treatment of peptic ulcer. The metabolism of PPI in the early market is affected by CYP2C19 gene polymorphism, and individual differences are large, and it cannot be used with CYP2C19 enzyme substrate drugs such as Clopidogrel. On the one hand, multidrug patients need PPIs with a lower risk of drug interaction; On the other hand, special patients need PPIs with less burden on the kidneys. Unlike other PPIs, Anaprazole possesses a dual-channel excretion mechanism through both the intestines and kidneys, reducing the drug’s retention time in the body and potentially decreasing toxic side effects (Liu et al., 2023). This characteristic makes it a safer option for patients with renal insufficiency and the elderly. In addition, Anaprazole sodium is less affected by CYP enzyme gene polymorphism, which is conducive to stable efficacy and improve safety.

With the ongoing reform of China’s healthcare system, the rational use of drugs, encompassing safety, effectiveness, and economic considerations, is becoming increasingly critical. Anaprazole, an independently developed PPI in China, has demonstrated favorable safety and symptom relief effects in clinical trials. This study aims to assess the economic viability of Anaprazole and Ilaprazole for treating DU, providing economic insights and support for clinical decision-making. A pharmacoeconomic evaluation was conducted from the healthcare system perspective, focusing on DU patients to identify a more cost-effective treatment strategy without compromising efficacy. The findings revealed that the ulcer healing rate in the Anaprazole group was similar to that in the Ilaprazole group. Additionally, the incremental cost-utility ratio (ICUR) for Anaprazole was below the willingness-to-pay threshold (WTP), suggesting that Anaprazole may offer greater clinical value in DU treatment than Ilaprazole. Given its favorable safety profile, efficacy, and the economic considerations of medical insurance, Anaprazole demonstrates superior clinical value. In conclusion, Anaprazole provides significant clinical efficacy, cost-efficiency, and enhanced economic benefits in DU treatment. Its inclusion in the NRDL minimally impacts the total health insurance expenditure, and the effect on the health insurance fund could be further mitigated through negotiated reductions in the prices of health insurance-covered drugs in exchange for volume increases.

There are several limitations in the methodology of this study. First, due to the absence of direct head-to-head clinical trials comparing Anaprazole and Ilaprazole, this study relied on a multicenter RCT comparing Anaprazole with Rabeprazole, and another RCT comparing Rabeprazole with Ilaprazole. This method of indirect comparison may introduce bias and limit the generalizability of the findings. Second, the MAIC analysis included a limited number of participants, which not only weakened the statistical power but also restricted the extrapolation of the results (Hatswell et al., 2020). To address these limitations, further analysis and long-term head-to-head comparative trials between Anaprazole and Ilaprazole are necessary to provide more reliable evidence. Additionally, Anaprazole and Ilaprazole use CYP3A4 as the main metabolic enzyme, both of which belong to the new generation of PPI drugs. However, due to the short launch time of the new generation of PPI drugs, the safety and effect data under long-term use may be relatively insufficient. However, how to translate the value of innovation into clinical value needs more long-term data support. Future studies may need to systematically evaluate the safety, effectiveness and economy of all PPI drugs in the treatment of duodenal ulcer, so as to provide a richer and more reliable evidence-based basis for clinical decision-making.

## Data Availability

The original contributions presented in the study are included in the article/Supplementary material, further inquiries can be directed to the corresponding authors.
